# Major Depressive Disorder Associated With Reduced Cortical Thickness in Women With Temporal Lobe Epilepsy

**DOI:** 10.3389/fneur.2019.01398

**Published:** 2020-01-17

**Authors:** Mateus Henrique Nogueira, Luciana Ramalho Pimentel da Silva, José Carlos Vasques Moreira, Thiago Junqueira Ribeiro de Rezende, Tamires Araújo Zanão, Brunno Machado de Campos, Clarissa Lin Yasuda, Fernando Cendes

**Affiliations:** ^1^Laboratory of Neuroimaging, Department of Neurology, University of Campinas – UNICAMP, Campinas, Brazil; ^2^The Brazilian Institute of Neuroscience and Neurotechnology – BRAINN, University of Campinas – UNICAMP, Campinas, Brazil; ^3^Laboratory of Medical Physics, University of Campinas – UNICAMP, Campinas, Brazil

**Keywords:** mesial temporal lobe epilepsy, major depressive disorder, women with epilepsy, cortical thickness abnormalities, surfaced-based methods

## Abstract

**Background:** Major Depressive Disorder (MDD) is highly prevalent in patients with mesial temporal lobe epilepsy (MTLE), especially in women, carrying significant morbidity. This study aimed to investigate the cortical thickness (CT) abnormalities associated with MDD in women with MTLE and hippocampal atrophy (HA). Also, we investigated the impact of MDD upon the volumes of the hippocampus and amygdala in these patients.

**Methods:** We included 50 women with MTLE and HA (20 left, LMTLE; 30 right, RMTLE), 41 healthy women in the control group, and 15 women with MDD without epilepsy. MTLE patients were subdivided into three groups: *MTLE-without-MDD* (23 MTLE patients without MDD), *MTLE-mild-MDD* (nine MTLE patients with mild symptoms of MDD), and *MTLE-severe-MDD* (18 MTLE patients with moderate to severe symptoms of MDD). The five groups were balanced for age (*p* = 0.56). All participants had high-resolution 3D T1-weighted images in a 3T scanner. We used FreeSurfer 6.0 for volumetry and CT parcellation. All participants were submitted to a clinical psychological evaluation through the Structured Clinical Interview for DSM-IV (SCID-IV) and completed the Beck Depression Inventory (BDI-II).

**Results:** We identified a smaller ipsilateral amygdala volume (*p* = 0.04) in the *MTLE-severe-MDD* group when compared to the control group. Our results presented a reduced ipsilateral lateral orbitofrontal cortex (*p* = 0.02) in the *MTLE-severe-MDD* in comparison to the *MTLE-mild-MDD* group. We also identified a thinner ipsilateral fusiform gyrus (*p* < 0.01) in the *MTLE-severe-MDD* compared to both *MTLE-without-MDD* and control groups. A reduced CT of the contralateral superior frontal gyrus (*p* = 0.02) was observed in the *MTLE-severe-MDD* in comparison to the *MTLE-mild-MDD* group.

**Conclusions:** The identification of areas with reduced CT and atrophy of the ipsilateral amygdala in women with MTLE and MDD suggest that the cortical thinning in the network of the paralimbic system is related to the co-occurrence and intensity of depressive symptoms in this group.

## Introduction

Major depressive disorder (MDD) has a high prevalence (20–55%) (1–6) in patients with mesial temporal lobe epilepsy (MTLE) (1, 7), in comparison to the general population (5–17%) (8). This psychiatric comorbidity brings significant concerns about the poor quality of life of these patients (2, 9). Also, the risk of suicide is a serious concern in patients with MTLE and MDD (13.5% of all suicides in people with epilepsy) (1, 10).

Although controversial, some studies have reported that women with or without epilepsy appear to be more affected by MDD than men (11). MDD has a female/male risk ratio of ~2:1 in the general population and is one of the primary disease-disability impairments among women around the world (12). Some authors (12) suggest that the occurrence of this psychiatric disorder is not related to sexual hormones; however, more studies are needed to clarify the interplay between biological susceptibility and environmental influences, including the social aspects.

In the last decades, neuroimaging studies have been attempting to identify neuro-anatomical substrates of MDD (13). The surface-based methods (14) engender accurate maps of cortical thickness (CT) and have provided a large amount of information about automatic brain segmentation, allowing comparisons among groups of patients and controls (13). Taking into account the CT abnormalities in patients with MDD, some studies have reported alterations in the paralimbic circuitry, which includes the orbitofrontal cortex (13), cingulate cortex (13), insula, temporal (15), and prefrontal regions (13, 16, 17). Furthermore, volumetric alterations of the subcortical structures, including the amygdala (18) and hippocampus (19–21), have been consistently reported in patients with epilepsy and psychiatric disorders.

Although there are relatively few studies evaluating cortical abnormalities in patients with MDD, there are even less of such studies in MTLE patients with MDD (22). Authors suggest a bidirectional interaction between epilepsy and MDD, not as a causal relationship, but perhaps due to yet unclear common pathogenic mechanisms involving similar structures in both MTLE and MDD (6). Further investigations are needed to clarify the profile and pathogenesis effects of concurrent MDD in patients with MTLE, and whether these patients are neurobiologically different from people with MDD without epilepsy (22).

We aimed to investigate the CT abnormalities associated with MDD in women with MTLE and unilateral hippocampal sclerosis (HS) (23). Also, we investigated the impact of MDD upon the volumes of the hippocampus and amygdala.

## Materials and Methods

### Subjects Selection

We evaluated 70 women with MTLE and a mean age of 45.7 [standard deviation (SD) ±8.9] years, currently followed at our outpatient epilepsy clinic (Tertiary Hospital—University of Campinas, UNICAMP, São Paulo, Brazil). During the analysis, we excluded four patients with bilateral HS, five patients with apparently non-lesional focal epilepsy, five patients with other brain lesions, two patients with MRI artifacts, and four patients due to errors in the automated cortex segmentation. The final sample included 50 women with MTLE [mean age of 43.9 (±8.8) years] with unilateral HS (left MTLE, *n* = 20; right MTLE, *n* = 30). All MTLE patients (age range from 26 to 64 years old) were diagnosed according to the International League Against Epilepsy (ILAE) criteria (24, 25) and had not been submitted to surgery. The definition of unilateral HS was made by an evaluation of an MRI epilepsy protocol by imaging experts as detailed previously (23, 26). We also included 15 women [38.8 (±9.7) years] with MDD without epilepsy (*MDD-without-epilepsy* group), and 41 healthy women without depressive symptoms as the control group [43.1 (±12.3) years].

### Clinical and Sociodemographic Data

In addition to age and gender, we collected clinical data from medical charts, including the age of onset, duration of epilepsy, monthly frequency of seizures, pharmacoresistance status, side of HS, and current antiepileptic and antidepressants drugs. None of the patients were taking levetiracetam (27). All patients in this study had normal average IQ. All the subjects signed the consent form approved by the University Ethics Committee before their admission to our study.

### Psychiatric Assessment

Participants were submitted to a clinical psychological interview with the Clinical Interview for DSM-IV (SCID-I) (28), focusing on possible current and past axis I psychiatric diagnoses. Additionally, we assessed depression symptoms by Beck Depression Inventory (BDI-II) (29). We followed recommended BDI-II cutoffs for the Brazilian population to determine MDD symptoms severity (0–13 as no depression, 14–19 as mild depression, 20–28 as moderate depression, and 29–63 as severe depression) (29). We only included patients with MDD diagnoses and excluded all subjects with other psychiatric comorbidities, as detailed in a previous study (3).

The 50 MTLE patients were subdivided into three groups according to the psychiatric assessment and BDI-II scores: *MTLE-without-MDD* (MTLE without depressive symptoms, *n* = 23), *MTLE-mild-MDD* (mild depressive symptoms, *n* = 9), and *MTLE-severe-MDD* (moderate to severe depressive symptoms, *n* = 18).

### MRI Acquisition and Cortical Thickness Analysis

All participants underwent a high-resolution volumetric T1-weighted MRI in a 3T scanner with the following protocol (30):

- Volumetric (3D) images acquired in the sagittal plane: T1-weighted image: isotropic voxels of 1 mm, acquired in the sagittal plane (1-mm-thick, no gap, flip angle = 8°, TR = 7.0 ms, TE = 3.2 ms, matrix = 240 × 240, FOV = 240 × 240 mm).

All images were visually checked for abnormalities unrelated to MTLE and motion artifacts.

For the CT and the analyses of the subcortical structures, we used the fully automated software FreeSurfer 6.0 (31, 32) (https://surfer.nmr.mgh.harvard.edu), which performed cortical reconstruction and volumetric segmentation. In summary, FreeSurfer corrects images by inhomogeneity of the magnetic field, aligns the images to the atlas of Talairach-Tournoux, removes non-cerebral tissue, segments gray matter, white matter, cerebrospinal fluid, and identifies voxels by the intensity of each element and its adjacent regions. Algorithms and a smoothing process are applied to correct topological defects (31, 32). A second smoothing interaction was used to identify the pial surface, which is segmented into small neuroanatomic regions, according to an atlas proposed by Desikan et al. (33). This automated labeling system subdivides the human cerebral cortex into 34 cortical regions of interest (ROIs) in each cerebral hemisphere, totaling 68 areas (33).

We performed a visual inspection of every individual processed image to guarantee a high pattern of quality and accuracy in the automated segmentation process. Brain regions with segmentation failure were excluded from our analysis.

We defined the hippocampus and the amygdala as subcortical structures of interest, considering their roles in both MTLE and the limbic system (34). To determine the ipsilateral hippocampus of the *MDD-without-epilepsy* and in the control group, we randomly assigned the hippocampal volume to follow the same proportion of HS lateralization of the MTLE patients. Accordingly, we determined that in 40% of these participants, the ipsilateral cerebral hemisphere was the left side, and consequently, in the remaining 60%, the right side was set as ipsilateral.

### Data Analysis

We performed the Kolmogorov-Smirnov test to evaluate data distribution and model fit and Pearson correlation tests to explore the relationship between continuous variables. To test group differences, we used the general linear model (GLM) with Sidak as *post-hoc* tests or Kruskal-Wallis test, when appropriate. Categorical variables were analyzed with the Pearson χ^2^-test. All the analyses considered the following groups: *MTLE-without-MDD, MTLE-mild-MDD, MTLE-severe-MDD, MDD-without-epilepsy*, and control.

In details, the analyses were performed as follows:

1. Comparison of the clinical and sociodemographic data among the groups;
2. Comparison of the hippocampi and amygdala volumes among the groups, including age, supratentorial volumes, and antidepressant drug usage as covariates. The effects of the age of onset of epilepsy and seizure frequency on both hippocampi/amygdala values of MTLE patients were also controlled in a separate analysis using multiple linear regression residuals. In addition, we conducted a correlation analysis among the hippocampi and amygdala volumes with BDI-II scores.
3. Correlation analysis among the 68 CT regions (34 ipsilateral/34 contralateral) and the BDI-II scores in the *MDD-without-epilepsy* group; this initial investigation was performed to establish a baseline of the CT analysis with the areas most associated with symptoms of depression. Since our analyses were exploratory and intended to guide the next steps (see item 4), we did not correct for the number of ROIs evaluated.
4. Subsequently, we selected the CT regions with significant correlation from the previous step [*p* < 0.05 and the absolute *r*-value of at least 0.5 (starting at a moderate correlation)] to perform comparisons among the MTLE groups, including age and antidepressant drug usage as nuisance covariates. As step two, we also controlled the effects of the age of onset of epilepsy and seizure frequency on CT values of MTLE patients in a separate analysis using multiple linear regression residuals. Ipsilateral and contralateral ROIs were analyzed in separated GLMs to avoid multicollinearity.

We reported the results using mean ± SD for parametric data and median (range) for data with non-parametric distribution. We used the Statistical Package for the Social Sciences—SPSS22 (Armonk, NY, U.S.A) to perform statistical analysis with a significant level set at *p* < 0.05.

## Results

### Clinical and Sociodemographic Information and BDI-II Scores

The *MTLE-without-epilepsy, MTLE-mild-MDD, MTLE-severe-MDD, MDD-without-MTLE*, and control groups were balanced for age (*p* = 0.56). Clinical and sociodemographic characteristics of the participants are presented in Table 1. We found a significant difference among the MTLE groups when we compared the frequency of seizures [Kruskal-Wallis test, χ^2^ (2, *N* = 50) = 41.8, *p* < 0.001]. The group *MTLE-severe-MDD* presented a higher frequency of seizures (*p* < 0.01) when compared to the *MTLE-mild-MDD* and *MTLE-without-MDD* groups. The usage of antidepressant drugs was significantly higher [χ^2^ (4, *N* = 106) = 66.5, *p* < 0.01] in the *MTLE-mild-MDD, MTLE-severe-MDD*, and *MDD-without-epilepsy* when compared to the *MTLE-without-MDD* and control groups. The groups *MTLE-mild-MDD, MTLE-severe-MDD*, and *MDD-without-epilepsy* did not present significant differences [χ^2^ (3, *N* = 42) = 1.01, *p* = 0.61] related to the antidepressant drug usage. As expected, the *MTLE-mild-MDD, MTLE-severe-MDD*, and *MDD-without-epilepsy* groups had higher scores on BDI-II [Kruskal-Wallis test, χ^2^ (4, *N* = 106) = 78.6, *p* < 0.001] when compared to the *MTLE-without-MDD* and control groups.

**Table 1:** Clinical and sociodemographic characteristics and BDI-II scores of the participants included in our study.

**Groups**	***MTLE-without-MDD N* = 23 mean (SD), or median (range), or *N* (%)**	***MTLE-mild-MDD N* = 9 mean (SD), or median (range), or *N* (%)**	***MTLE-severe-MDD N* = 18 mean (SD), or median (range), or *N* (%)**	***MDD-without-epilepsy N* = 15 mean (SD), or median (range), or *N* (%)**	***Control N* = 41 mean (SD), or median (range), or *N* (%)**	***p-*value**
Age (years)	44.9 (±8.1)	43.3 (±11.1)	43.1 (±9.2)	38.9 (±9.8)	43.1 (±12.3)	0.56
Duration of epilepsy	31.7 (±12.3)	31.3 (±13.9)	30.7 (14.4)	NA	NA	0.97
Age of onset	12 (1–37)	4 (1–32)	5 (1–48)	NA	NA	0.46
Side of hippocampal atrophy				NA	NA	0.86
Left	10 (43.5%)	3 (33.3%)	7 (38.9%)			
Right	13 (56.6%)	6 (66.7%)	11 (61.1%)			
Seizure frequency (monthly)	0.5 (0–12)	0.5 (0–4)	3.5 (0–12)	NA	NA	<0.001
Pharmacoresistance				NA	NA	0.41
Yes	12 (52.2%)	5 (55.6%)	13 (72.2%)			
No	11 (47.8%)	4 (44.4%)	5 (27.8%)			
Antiepileptic drugs				NA	NA	0.48
Monotherapy	6 (26.1%)	4 (44.4%)	4 (22.2%)			
Polytherapy	17 (73.9%)	5 (55.6%)	14 (77.8%)			
Antidepressant drugs						<0.01
Yes	2 (8.7%)	6 (66.7%)	15 (83.3%)	12 (80%)	0 (0%)	
No	21 (91.3%)	3 (33.3%)	3 (16.7%)	3 (20%)	41 (100%)	
BDI scores	3 (0–9)	15 (12–19)	26 (20–40)	30 (10–41)	4 (0–9)	<0.01

*MTLE, mesial temporal lobe epilepsy; MDD, Major Depressive Disorder; MTLE-without-MDD, MTLE patients without psychiatric disorders; MTLE-mild-MDD, MTLE patients with mild depressive symptoms; MTLE-severe-MDD, MTLE patients with moderate to severe depressive symptoms; MDD-without-epilepsy, patients with Major Depressive Disorder without epilepsy; N, number of participants; SD, standard deviation; BDI, Beck Depression Inventory; NA, not applicable*.

### Subcortical Analysis

**Figure 1:**
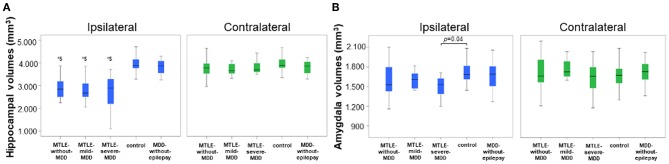
Volumes of hippocampus and amygdala according to the groups. **(A)** Ipsilateral and contralateral hippocampus volumes. The *MTLE-without-MDD, MTLE-mild-MDD, and MTLE-severe-MDD groups* (*$) presented smaller ipsilateral hippocampus (*p* < 0.01) when compared to the *MDD-without-epilepsy* and control groups. **(B)** Ipsilateral and contralateral amygdala volumes according to the groups. The *MTLE-severe-MDD* group presented a smaller ipsilateral amygdala (*p* = 0.04) when compared to the control group. MTLE, mesial temporal lobe epilepsy; MDD, Major Depressive Disorder; *MTLE-without-MDD*, MTLE patients without depression; *MTLE-mild-MDD*, MTLE patients with mild symptoms of depression; *MTLE-severe-MDD*, MTLE patients with moderate to severe symptoms of depression; *MDD-without-epilepsy*, patients without epilepsy with MDD.

We compared both hippocampus and amygdala volumes among the groups. There was a significant multivariate group effect in the ipsilateral analyses [*F*_(8, 186)_ = 5.26, *p* < 0.001; Pillai's Trace = 0.37; η^2^ = 0.18]. As expected, the *MTLE-without-MDD, MTLE-mild-MDD*, and *MTLE-severe-MDD* groups presented a smaller ipsilateral hippocampus [*F*_(4, 93)_ = 8.56, *p* < 0.01, partial η^2^ = 0.27] when compared to the control group, however, they did not differ (*p* > 0.05) from the *MDD-without-epilepsy* group. No significant differences in the contralateral hippocampus were observed among all groups [*F*_(4, 95)_ = 0.84, *p* = 0.5, partial η^2^ = 0.03], as presented in Figure 1A. Taking into account the amygdala, we only observed a significant reduction in the volume of the ipsilateral amygdala in the *MTLE-severe-MDD* group [*F*_(4, 93)_ = 2.8, *p* = 0.04, partial η^2^ = 0.11] when compared to the control group, as shown in Figure 1B. No difference was observed in the contralateral amygdala volume among the groups [*F*_(4, 95)_ = 1.31, *p* = 0.27, partial η^2^ = 0.05]. We did not observe significant differences in the correlation analysis (*r* < −0.5, *n* = 106, *p* > 0.05, one-tailed) among the hippocampus and amygdala volumes with the BDI-II scores in the five groups. We conducted further analysis considering only the MTLE groups and the subdivision of left and right atrophy sides. However, no significant differences were detected.

### Cortical Thickness Correlations

In the first step, we investigated the significant correlations between CT and BDI-II scores in patients with depression without epilepsy (*MDD-without-epilepsy* group). We found 24 CT areas with significant negative correlations with BDI-II scores in the *MDD-without-epilepsy* group, as shown in Table S1.

### Cortical Thickness Group Comparisons

**Figure 2:**
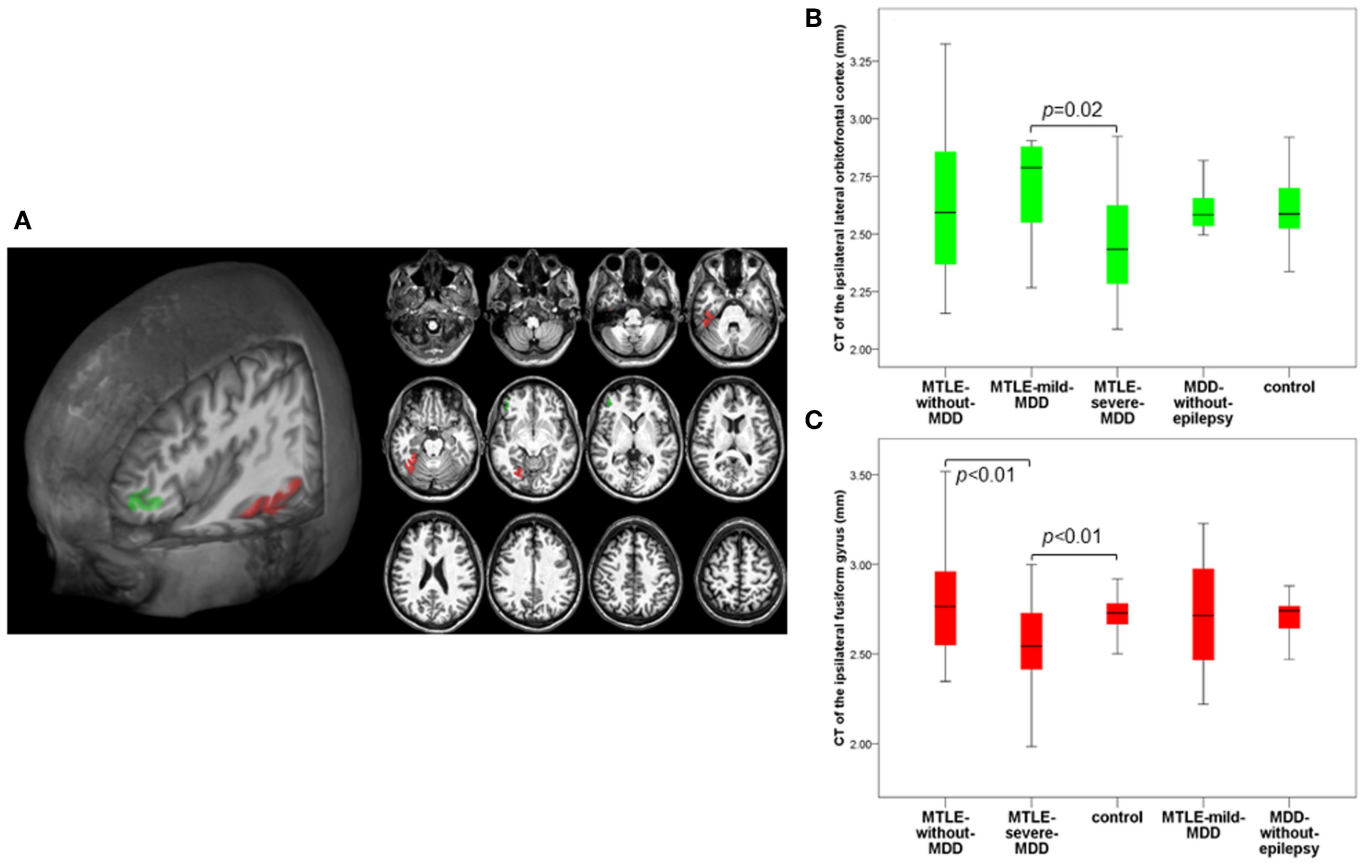
Composition panel illustrating CT reduction in the ipsilateral lateral orbitofrontal cortex (green) and in the ipsilateral fusiform gyrus (red). **(A)** 3D and 2D automated FreeSurfer segmentation of the ipsilateral lateral orbitofrontal cortex and the ipsilateral fusiform gyrus. **(B)** Cortical thickness abnormalities in the ipsilateral lateral orbitofrontal cortex according to the groups. **(C)** Cortical thickness abnormalities in the ipsilateral fusiform gyrus according to the groups. CT, cortical thickness; MTLE, mesial temporal lobe epilepsy; MDD, Major Depressive Disorder; *MTLE-without-MDD*, MTLE patients without depression; *MTLE-mild-MDD*, MTLE patients with mild symptoms of depression; *MTLE-severe-MDD*, MTLE patients with moderate to severe symptoms of depression; *MDD-without-epilepsy*, patients without epilepsy with MDD.

**Figure 3:**
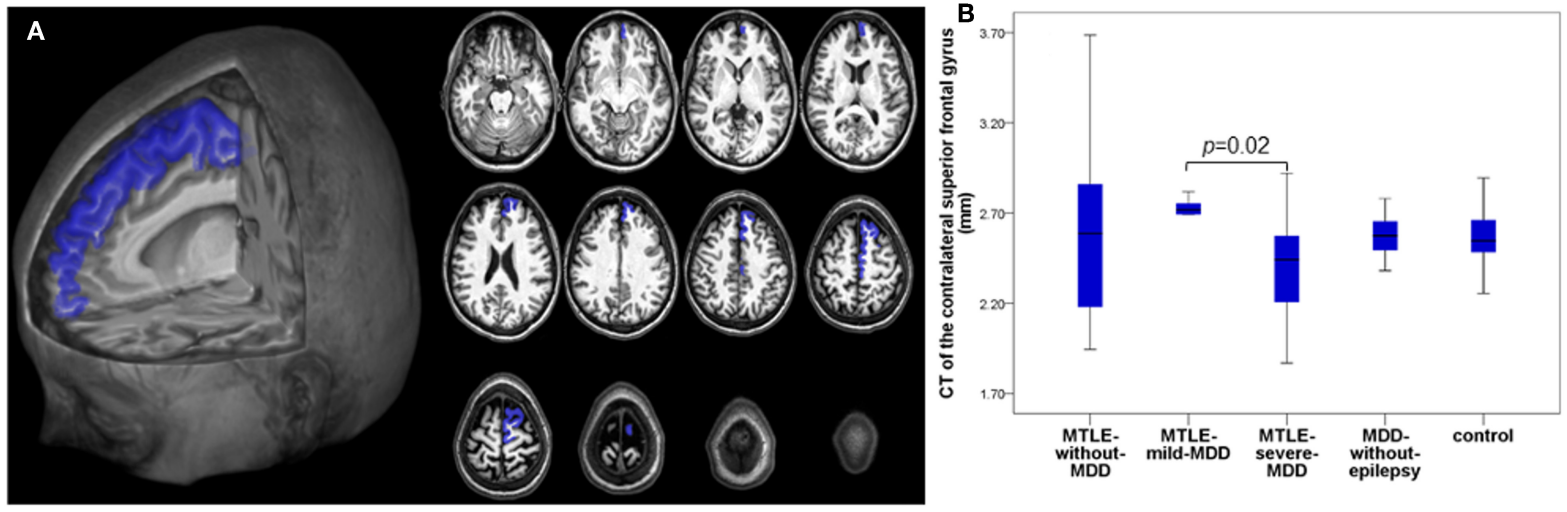
Composition panel illustrating CT reduction in the contralateral superior frontal gyrus (blue). **(A)** 2D and 3D automated FreeSurfer segmentation of the contralateral superior frontal gyrus. **(B)** CT of the contralateral superior frontal gyrus among the groups. CT, cortical thickness; MTLE, mesial temporal lobe epilepsy; MDD, Major Depressive Disorder; *MTLE-without-MDD*, MTLE patients without depression; *MTLE-mild-MDD*, MTLE patients with mild symptoms of depression; *MTLE-severe-MDD*, MTLE patients with moderate to severe symptoms of depression; *MDD-without-epilepsy*, patients without epilepsy with MDD.

As planned, we performed group comparisons with the 24 CT regions (16 CT ipsilateral and eight CT contralateral regions) significantly associated with symptoms of depression in our *MDD-without-epilepsy* group. The multivariate analysis of CT among the five groups (*MTLE-without-MDD, MTLE-mild-MDD, MTLE-severe-MDD, MDD-without-epilepsy*, and control) was significant for the ipsilateral [*F*_(64, 296)_ = 1.46, *p* = 0.02; Pillai's Trace = 0.96; η^2^ = 0.24] and for the contralateral regions [*F*_(32, 340)_ = 1.49, *p* = 0.047; Pillai's Trace = 0.49; η^2^ = 0.12]. We observed a reduced CT of the ipsilateral lateral orbitofrontal cortex [*F*_(4, 86)_ = 0.52, *p* = 0.02, partial η^2^ = 0.13] in the *MTLE-severe-MDD* when compared to the *MTLE-mild-MDD* group. A thinner ipsilateral fusiform gyrus [*F*_(4, 86)_ = 0.52, *p* < 0.01; partial η^2^ = 0.16] was found in the *MTLE-severe-MDD* when compared to the *MTLE-without-MDD* and control groups, as presented in Figure 2. We noticed a reduced CT of the contralateral superior frontal gyrus [*F*_(4, 88)_ = 0.67, *p* = 0.02, partial η^2^ = 0.15] in the *MTLE-severe-MDD* when compared to the *MTLE-mild-MDD* group (Figure 3).

## Discussion

The application of a semi-structured psychological clinical interview (SCID-I) in patients with MTLE allowed us to diagnose those with and without depression. The analysis of CT atrophy in these groups revealed a differential pattern of CT abnormalities in the MTLE subgroups with and without depression. In addition, atrophy of the ipsilateral amygdala was detected exclusively in the *MTLE-severe-MDD* group. Differently from previous hypothesis-driven studies, we initially searched for brain regions associated with the degree of depressive symptoms in the *MDD-without-epilepsy* group. This procedure allowed us to reduce the number of variables appropriately and select a more specific subset of cortical areas for data-driven analysis. This approach allowed us to characterize a unique pattern of cortical alterations in the *MTLE-severe-MDD* group, including the lateral orbitofrontal cortex, fusiform, and superior frontal gyrus.

Psychiatric comorbidities, especially MDD, constitute a significant source of concerns in pharmacoresistant epilepsy, as previously reported (3, 35). The course of MTLE can be impacted by psychiatric aspects, including the predisposition to seizure worsening (3, 36), unsatisfactory response to pharmacologic treatment (37), AEDs tolerance, and the surgical outcome (35, 36). One study (38) included 780 consecutive patients with recently diagnosed epilepsy and identified significant psychiatric comorbidities preceding onset. MDD history had twice the risk of pharmacoresistance. Another study (39) evaluating 100 TLE patients who underwent anterior temporal lobectomy reported a lifetime history of MDD in 12% of seizure-free patients (after 2 years of the surgical procedure) in comparison to MDD history in 79% of patients who persisted with disabling seizures. These studies emphasize the negative interaction between MTLE and MDD, highlighting the possible involvement of common pathogenic mechanisms in both disorders (6).

Patients with psychiatric diagnoses have a higher risk of presenting refractory seizures (3, 40). In our study, the *MTLE-severe-MDD* group showed a higher frequency of seizures when compared to the *MTLE-without-MDD* and *MTLE-mild-MDD* groups. A recent study (27) evaluated 933 patients with epilepsy to identify the prevalence of depression, taking into account some factors as AEDs, seizure frequency, and other clinical and sociodemographic data. They found a significant association between the seizure frequency and the number of AEDs prescribed, with the occurrence of depression in patients with epilepsy. Patients with pharmacoresistant-epilepsy presented more severe symptoms of depression when compared to seizure-free patients.

Our results demonstrated a significant volume reduction in the ipsilateral amygdala in the *MTLE-severe-MDD* compared to the control group. Although symptoms of depression have been related to changes in both the amygdala and hippocampus (41), the MTLE groups were undistinguished in terms of HS. Both regions are associated with the modulation of emotional behavior and motivation, and they have connections to the orbitofrontal cortex, medial prefrontal cortex, and hypothalamic areas (41).

The amygdala has a crucial role in emotional memory and perception (42), as well as being highly associated with the genesis and spreading of epileptiform activity in MTLE. It also plays an essential role in psychiatric symptoms in pharmacoresistant epilepsy (41). Taking into account the connections between the amygdala and the brainstem regions, some studies (43) have reported abnormalities in emotion recognition related to amygdala dysfunction in MTLE patients. Very few studies analyzed CT in adult patients with epilepsy and MDD. One study (44) evaluated subcortical and cortical differences in 88 children with epilepsy, and 25 of these children had a current anxiety disorder. They showed a larger left amygdala in the group of children with anxiety disorder, as well as thinning in the left medial orbitofrontal cortex, right lateral orbitofrontal, and in the right frontal pole in this group. Given the importance of the amygdala in both epilepsy and psychiatric symptoms, further research (especially in MTLE patients) should assess the impact of antidepressants and AEDs usage, mainly focusing on mood stabilizers concerning the dynamic changes processes of the amygdala.

Although fewer studies have previously shown a correlation between hippocampal volumes and the BDI-II scores, we did not observe such association in our analyses. One study (20) evaluated the relationship between depression and hippocampal volume loss in 55 patients with TLE, showing that patients with right TLE and depression presented a reduced left hippocampal volume. They concluded that the observed contralateral hippocampal atrophy could not be exclusively attributed to epilepsy, suggesting a significant impact of the depression on the hippocampal volume loss. A previous study of our group (21) used voxel-based morphometry to investigate the differences in gray matter volume in 48 MTLE patients with and without depression (compared to 96 healthy controls). There was widespread gray matter atrophy in MTLE patients with MDD, but no correlation between BDI scores and regional gray matter atrophy. More studies are necessary to better clarify this relationship between subcortical structures and the intensity of depression symptoms in patients with MTLE.

Our results revealed CT abnormalities in the ipsilateral orbitofrontal cortex, ipsilateral fusiform gyrus, and in the contralateral superior frontal gyrus in the group of patients with concurrent MTLE and moderate to severe symptoms of MDD. Some studies have focused on depression-associated abnormalities in frontal regions related to emotional regulation (45), including the dorsolateral prefrontal cortex, anterior cingulate areas (46), and the orbitofrontal cortex (22, 47). Unfortunately, while several studies have examined structural alterations (with both gray and white matter) in TLE, fewer have accurately analyzed the structural changes in MTLE patients with concurrent MDD. Further investigation is still required to achieve a better understanding of the relationship between alterations of extratemporal and frontal regions in MTLE patients with MDD (22).

A considerable number of functions have been attributed to the orbitofrontal cortex, such as driving social behavior, inhibiting responses, emotional and reward of decision-making, among others (48, 49). The orbitofrontal cortex connects bidirectionally with the sensory association cortices and temporal lobe areas, having a robust connection with the amygdala (46) and being associated with the modulation of the aggressive behavior (50). Hypometabolism in the orbitofrontal cortex was identified in patients with TLE and MDD, suggesting anomalies in the functioning of glia and neurons of this region (22, 51). In line with previous studies on depression, we observed a negative association between the intensity of MDD and CT of the lateral orbitofrontal cortex in patients with MTLE. The most extensive worldwide study (13) (ENIGMA-MDD) evaluated the cortical structural alterations in depression in 2,148 patients with MDD compared to 7,957 healthy controls. A reduced bilateral CT in the orbitofrontal cortex, insula, temporal poles, and cingulate (anterior and posterior) regions was associated with MDD in adults. In another study, the correlation between the orbitofrontal cortex CT and depressive symptoms scores in 38 patients with TLE and 45 controls (22) demonstrated a negative correlation in controls and a positive correlation in TLE patients. They also detected a positive correlation between the BDI-II scores and the right fusiform gyrus, and a negative correlation with a small region in the right parietal cortex. One limitation of that study was the small number of TLE patients, added to the absence of a structured diagnostic interview for MDD. In the present study, we applied a structured diagnostic interview and used different methodological approaches for neuroimaging analysis, taking into account the lateralization of ipsilateral and contralateral structures, in contrast to the left and right side of the brain regions.

Atrophy of the fusiform gyrus was present in the *MTLE-severe-MDD* group, compared to the *MTLE-without-MDD* and control groups. The fusiform gyrus, or lateral occipitotemporal gyrus, is associated with the processing of color information, face and body recognition, word recognition, and within-category identification (52). Donix et al. (53) evaluated 27 young individuals with MDD and 23 older participants without MDD and demonstrated an association between the fusiform cortices and subjective memory impairments in the young group with MDD. Another study (54) investigated whether the CT abnormalities indicate initial adverse properties of environmental and genetic risk factors predisposing MDD or appear with the mood disorders onset. They evaluated MRI data from 111 young adults without MDD but with a high familial risk to develop this psychiatric disorder and 93 healthy controls. Reduction in the fusiform thickness and the right parahippocampal gyrus was associated with a familial vulnerability to mood disorders. Although these studies enrolled patients without epilepsy, their results support our findings as they indicate a significant role of the atrophic fusiform gyrus in MDD. The ENIGMA-Epilepsy consortium (55) analyzed CT from 754 MTLE patients, regardless of the presence of depressive symptoms and confirmed reduced CT of the ipsilateral fusiform and the superior frontal gyrus (among other regions) in patients with left MTLE. The similarity between those findings (in isolated MDD and epilepsy) and ours (concurrent MTLE and MDD) reinforce the hypothesis of shared physiopathology for depression and MTLE.

In agreement with the comprehensive examination of CT performed in the ENIGMA-MDD study (13), we also observed superior frontal gyrus atrophy in the *MTLE-severe-MDD* compared to the *MTLE-mild-MDD* group. However, while the ENIGMA-MDD study (13) demonstrated reduced surface area exclusively in adolescents with depression (without alterations in CT), we identified reduced CT in the same region in our subgroup of adult patients. Conversely, another study (56) with 32 MDD patients (16 untreated and 16 first episode) examined the cortical maps of thickness, gyrification, and surface area, and reported increased surface area in the superior frontal regions without CT abnormalities. Since the function of the superior frontal gyrus is related both to the self-awareness in association with the sensory system (57) and to the “laughter brain region” (58), its involvement in the manifestation of depression is expected. This novel finding in our study and the controversies from the previous research related to superior frontal gyrus and depression highlights the need for further neuroimaging studies, including functional MRI, to investigate the impact of MDD on dysfunctions of the frontal lobe in epilepsy.

## Limitations

A reduced number of individuals and cross-sectional design was a limitation with possible impact in our statistical models; however, we applied corrections for our multiple comparisons to avoid false-positive results. Another relevant point was the selective inclusion of women in our study. This composition was determined because we only had women in our group with depression without epilepsy. The most likely explanation for this bias is that in our cultural scenario, men have been remarkably resistant to seek health care, especially mental health care. Moreover, our outpatient clinic is part of a neurological tertiary center, specialized in epilepsy care. The individuals with only depression were volunteers and recruited for transversal research, without implications or personal gain to their treatments. The participants who were not receiving any MDD treatment were referred to an adequate treatment service. Our results are preliminary, and further studies with a larger number of patients, including men, and validation in independent cohorts are necessary for confirming our findings.

## Conclusions

Our findings suggest a specific pattern of CT atrophy in women with MTLE and depression, implicating a dysfunction in networks composed of some structures related to both epilepsy and MDD. These observations contribute to the existing theory about the bidirectional interaction between epilepsy and depression. However, additional studies with a higher number of subjects (mixing men and women) are necessary to explore these abnormalities in epilepsy, with an investigation of other structural characteristics as well as a combination with functional analyses. The identification of specific alterations in patients with concurrent epilepsy and depression may provide future targets for personalized treatment of the two comorbidities.

## Data Availability Statement

All datasets generated for this study are included in the article/Supplementary Material. Any additional information can be available upon reasonable request.

## Ethics Statement

The studies involving human participants were reviewed and approved by Ethics Committee of the State University of Campinas (CEP/FCM 1191/2011). The patients/participants provided their written informed consent to participate in this study.

## Author Contributions

MN designed the study, recruited and evaluated patients, visually checked and analyzed the MRI images, performed statistical analysis, and wrote the paper. LP recruited patients, contributed to the discussion session, performed statistical analysis, and wrote the paper. JV performed the processing of the MRI and implemented the FreeSurfer scripts. TR contributed to the knowledge and discussion about the FreeSurfer methodology in addition to the creation of the FreeSurfer segmentation images to illustrate our results. TZ recruited and evaluated patients. BC created the FreeSurfer segmentation images to illustrate our results and contributed to the discussion session. CY and FC designed the study, performed statistical analysis, wrote the paper, and provided mentorship and funding for the study.

### Conflict of Interest

The authors declare that the research was conducted in the absence of any commercial or financial relationships that could be construed as a potential conflict of interest.

## Supplementary Material

The Supplementary Material for this article can be found online at: https://www.frontiersin.org/article/10.3389/fneur.2019.01398/full#supplementary-material.

## References

[1] Valente KD Busatto Filho G. Depression and temporal lobe epilepsy represent an epiphenomenon sharing similar neural networks: clinical and brain structural evidences. Arq Neuropsiquiatr. (2013) 71:183–90. 10.1590/S0004-282X2013000300011 23563720

[2] Gilliam F. Optimizing health outcomes in active epilepsy. Neurology. (2002) 58(8 Suppl. 5):S9–20. 10.1212/WNL.58.8_suppl_5.S9 11971128

[3] Nogueira MH Yasuda CL Coan AC Kanner AM Cendes F. Concurrent mood and anxiety disorders are associated with pharmacoresistant seizures in patients with MTLE. Epilepsia. (2017) 58:1268–76. 10.1111/epi.13781 28555776

[4] Bragatti JA Torres CM Londero RG Martin KC Souza AC Hidalgo MP. Prevalence of psychiatric comorbidities in temporal lobe epilepsy in a Southern Brazilian population. Arq Neuropsiquiatr. (2011) 69:159–65. 10.1590/S0004-282X2011000200003 21537552

[5] Araújo Filho GM Rosa VP Lin K Caboclo LO Sakamoto AC Yacubian EM Psychiatric comorbidity in epilepsy: a study comparing patients with mesial temporal sclerosis and juvenile myoclonic epilepsy. Epilepsy Behav. (2008) 13:196–201. 10.1016/j.yebeh.2008.01.008 18313989

[6] Kanner AM Scharfman H Jette N Anagnostou E Bernard C Camfield C. Epilepsy as a network disorder (1): what can we learn from other network disorders such as autistic spectrum disorder and mood disorders? Epilepsy Behav. (2017) 77:106–13. 10.1016/j.yebeh.2017.09.014 29107450 PMC9835466

[7] Kanner AM. Depression in epilepsy: prevalence, clinical semiology, pathogenic mechanisms, and treatment. Biol Psychiatry. (2003) 54:388–98. 10.1016/s0006-3223(03)00469-4 12893113

[8] Swinkels WA Kuyk J van Dyck R Spinhoven P. Psychiatric comorbidity in epilepsy. Epilepsy Behav. (2005) 7:37–50. 10.1016/j.yebeh.2005.04.012 15975853

[9] Boylan LS Flint LA Labovitz DL Jackson SC Starner K Devinsky O Depression but not seizure frequency predicts quality of life in treatment-resistant epilepsy. Neurology. (2004) 62:258–61. 10.1212/01.WNL.0000103282.62353.85 14745064

[10] Pompili M Girardi P Tatarelli R. Death from suicide versus mortality from epilepsy in the epilepsies: a meta-analysis. Epilepsy Behav. (2006) 9:641–8. 10.1016/j.yebeh.2006.06.019 17011240

[11] Gaus V Kiep H Holtkamp M Burkert S Kendel F Gender differences in depression, but not in anxiety in people with epilepsy. Seizure. (2015) 32:37–42. 10.1016/j.seizure.2015.07.012 26552559

[12] Kessler RC. Epidemiology of women and depression. J Affect Disord. (2003) 74:5–13. 10.1016/s0165-0327(02)00426-3 12646294

[13] Schmaal L Hibar DP Samann PG Hall GB Baune BT Jahanshad N. Cortical abnormalities in adults and adolescents with major depression based on brain scans from 20 cohorts worldwide in the ENIGMA Major Depressive Disorder Working Group. Mol Psychiatry. (2017) 22:900–9. 10.1038/mp.2016.60 27137745 PMC5444023

[14] Fischl B van der Kouwe A Destrieux C Halgren E Segonne F Salat DH. Automatically parcellating the human cerebral cortex. Cereb Cortex. (2004) 14:11–22. 10.1093/cercor/bhg087 14654453

[15] Lange C Irle E. Enlarged amygdala volume and reduced hippocampal volume in young women with major depression. Psychol Med. (2004) 34:1059–64. 10.1017/S0033291703001806 15554576

[16] Kempton MJ Salvador Z Munafo MR Geddes JR Simmons A Frangou S. Structural neuroimaging studies in major depressive disorder. Meta-analysis and comparison with bipolar disorder. Arch Gen Psychiatry. (2011) 68:675–90. 10.1001/archgenpsychiatry.2011.60 21727252

[17] Bora E Fornito A Pantelis C Yucel M. Gray matter abnormalities in Major Depressive Disorder: a meta-analysis of voxel based morphometry studies. J Affect Disord. (2012) 138:9–18. 10.1016/j.jad.2011.03.049 21511342

[18] Briellmann RS Hopwood MJ Jackson GD. Major depression in temporal lobe epilepsy with hippocampal sclerosis: clinical and imaging correlates. J Neurol Neurosurg Psychiatry. (2007) 78:1226–30. 10.1136/jnnp.2006.104521 17259350 PMC2117607

[19] Quiske A Helmstaedter C Lux S Elger CE. Depression in patients with temporal lobe epilepsy is related to mesial temporal sclerosis. Epilepsy Res. (2000) 39:121–5. 10.1016/S0920-1211(99)00117-5 10759300

[20] Shamim S Hasler G Liew C Sato S Theodore WH. Temporal lobe epilepsy, depression, and hippocampal volume. Epilepsia. (2009) 50:1067–71. 10.1111/j.1528-1167.2008.01883.x 19054394 PMC2692336

[21] Salgado PC Yasuda CL Cendes F. Neuroimaging changes in mesial temporal lobe epilepsy are magnified in the presence of depression. Epilepsy Behav. (2010) 19:422–7. 10.1016/j.yebeh.2010.08.012 20850388

[22] Butler T Blackmon K McDonald CR Carlson C Barr WB Devinsky O. Cortical thickness abnormalities associated with depressive symptoms in temporal lobe epilepsy. Epilepsy Behav. (2012) 23:64–7. 10.1016/j.yebeh.2011.10.001 22099527 PMC3259282

[23] Cendes F Sakamoto AC Spreafico R Bingaman W Becker AJ. Epilepsies associated with hippocampal sclerosis. Acta Neuropathol. (2014) 128:21–37. 10.1007/s00401-014-1292-0 24823761

[24] Berg AT Berkovic SF Brodie MJ Buchhalter J Cross JH van Emde Boas W. Revised terminology and concepts for organization of seizures and epilepsies: report of the ILAE Commission on Classification and Terminology, 2005-2009. Epilepsia. (2010) 51:676–85. 10.1111/j.1528-1167.2010.02522.x 20196795

[25] ILAE I Proposal for revised classification of epilepsies and epileptic syndromes. Commission on classification and terminology of the international league against epilepsy. Epilepsia. (1989) 30:389–99. 2502382 10.1111/j.1528-1157.1989.tb05316.x

[26] Coan AC Kubota B Bergo FP Campos BM Cendes F. 3T MRI quantification of hippocampal volume and signal in mesial temporal lobe epilepsy improves detection of hippocampal sclerosis. AJ NR Am J Neuroradiol. (2014) 35:77–83. 10.3174/ajnr.A3640 23868151 PMC7966486

[27] Joshi R Tripathi M Gupta P Goyal A Gupta YK. Depression in patients receiving pharmacotherapy for epilepsy: an audit in a tertiary care centre. Pharmacol Rep. (2019) 71:848–54. 10.1016/j.pharep.2019.04.021 31398575

[28] Del-Ben CM Vilela JAA Crippa JAS Hallak JEC Labate CM Zuardi AW Reliability of the structured clinical interview for DSM-IV-clinical version translated into Portuguese. Braz J Psychiatry. (2001) 23:156–9. 10.1590/S1516-44462001000300008

[29] Gomes-Oliveira MH Gorenstein C Lotufo Neto F Andrade LH Wang YP. Validation of the Brazilian Portuguese version of the Beck Depression Inventory-II in a community sample. Braz J Psychiatry. (2012) 34:389–94. 10.1016/j.rbp.2012.03.005 23429809

[30] de Campos BM Coan AC Lin Yasuda C Casseb RF Cendes F. Large-scale brain networks are distinctly affected in right and left mesial temporal lobe epilepsy. Hum Brain Mapp. (2016) 37:3137–52. 10.1002/hbm.23231 27133613 PMC5074272

[31] Reuter M Schmansky NJ Rosas HD Fischl B. Within-subject template estimation for unbiased longitudinal image analysis. Neuroimage. (2012) 61:1402–18. 10.1016/j.neuroimage.2012.02.084 22430496 PMC3389460

[32] Fischl B Dale AM. Measuring the thickness of the human cerebral cortex from magnetic resonance images. Proc Natl Acad Sci USA. (2000) 97:11050–5. 10.1073/pnas.200033797 10984517 PMC27146

[33] Desikan RS Segonne F Fischl B Quinn BT Dickerson BC Blacker D. An automated labeling system for subdividing the human cerebral cortex on MRI scans into gyral based regions of interest. Neuroimage. (2006) 31:968–80. 10.1016/j.neuroimage.2006.01.021 16530430

[34] Gaitatzis A Trimble MR Sander JW. The psychiatric comorbidity of epilepsy. Acta Neurol Scand. (2004) 110:207–20. 10.1111/j.1600-0404.2004.00324.x 15355484

[35] Kanner AM Lennox-lombroso lecture, 2013: psychiatric comorbidities through the life of the seizure disorder: a complex relation with a not so complex solution. Epilepsy Curr. (2014) 14:323–8. 10.5698/1535-7597-14.6.323 25678862 PMC4325585

[36] Tellez-Zenteno JF Patten SB Jetté N Williams J Wiebe S. Psychiatric comorbidity in epilepsy: a population-based analysis. Epilepsia. (2007) 48:2336–44. 10.1111/j.1528-1167.2007.01222.x 17662062

[37] Hesdorffer DC Hauser WA Annegers JF Cascino G. Major depression is a risk factor for seizures in older adults. Ann Neurol. (2000) 47:246–9. 10.1002/1531-8249(200002)47:2<246::AID-ANA17>3.0.CO;2-E 10665498

[38] Hitiris N Mohanraj R Norrie J Sills GJ Brodie MJ. Predictors of pharmacoresistant epilepsy. Epilepsy Res. (2007) 75:192–6. 10.1016/j.eplepsyres.2007.06.003 17628429

[39] Kanner AM Byrne R Chicharro A Wuu J Frey M. A lifetime psychiatric history predicts a worse seizure outcome following temporal lobectomy. Neurology. (2009) 72:793–9. 10.1212/01.wnl.0000343850.85763.9c 19255406

[40] Thompson NJ McGee RE Garcia-Williams A Selwa LM Stoll SC Johnson EK. The impact of a depression self-management intervention on seizure activity. Epilepsy Behav. (2019). 10.1016/j.yebeh.2019.106504. [Epub ahead of print]. 31648928 PMC7002270

[41] Yilmazer-Hanke D O'Loughlin E McDermott K. Contribution of amygdala pathology to comorbid emotional disturbances in temporal lobe epilepsy. J Neurosci Res. (2016) 94:486–503. 10.1002/jnr.23689 26525920

[42] Hamilton JP Siemer M Gotlib IH. Amygdala volume in major depressive disorder: a meta-analysis of magnetic resonance imaging studies. Mol Psychiatry. (2008) 13:993–1000. 10.1038/mp.2008.57 18504424 PMC2739676

[43] Monti G Meletti S. Emotion recognition in temporal lobe epilepsy: a systematic review. Neurosci Biobehav Rev. (2015) 55:280–93. 10.1016/j.neubiorev.2015.05.009 25999121

[44] Jones JE Jackson DC Chambers KL Dabbs K Hsu DA Stafstrom CE. Children with epilepsy and anxiety: subcortical and cortical differences. Epilepsia. (2015) 56:283–90. 10.1111/epi.12832 25580566 PMC4340751

[45] Pandya M Altinay M Malone DA Jr Anand A. Where in the brain is depression? Curr Psychiatry Rep. (2012) 14:634–42. 10.1007/s11920-012-0322-7 23055003 PMC3619732

[46] Koolschijn PC van Haren NE Lensvelt-Mulders GJ Hulshoff Pol HE Kahn RS. Brain volume abnormalities in major depressive disorder: a meta-analysis of magnetic resonance imaging studies. Hum Brain Mapp. (2009) 30:3719–35. 10.1002/hbm.20801 19441021 PMC6871089

[47] Koenigs M Huey ED Calamia M Raymont V Tranel D Grafman J. Distinct regions of prefrontal cortex mediate resistance and vulnerability to depression. J Neurosci. (2008) 28:12341–8. 10.1523/JNEUROSCI.2324-08.2008 19020027 PMC2644261

[48] Stalnaker TA Cooch NK Schoenbaum G. What the orbitofrontal cortex does not do. Nat Neurosci. (2015) 18:620–7. 10.1038/nn.3982 25919962 PMC5541252

[49] Sadacca BF Wikenheiser AM Schoenbaum G. Toward a theoretical role for tonic norepinephrine in the orbitofrontal cortex in facilitating flexible learning. Neuroscience. (2017) 345:124–9. 10.1016/j.neuroscience.2016.04.017 27102419 PMC5461826

[50] Numan M Neurobiology of Social Behavior:Toward an Understanding of the Prosocial and Antisocial Brain. Vol. 11 London; Waltham, MA: Elsevier/AP, Academic Press is an imprint of Elsevier (2015). p. 345.

[51] Salzberg M Taher T Davie M Carne R Hicks RJ Cook M. Depression in temporal lobe epilepsy surgery patients: an FDG-PET study. Epilepsia. (2006) 47:2125–30. 10.1111/j.1528-1167.2006.00860.x 17201712

[52] Grill-Spector K Weiner KS. The functional architecture of the ventral temporal cortex and its role in categorization. Nat Rev Neurosci. (2014) 15:536–48. 10.1038/nrn3747 24962370 PMC4143420

[53] Donix M Haussmann R Helling F Zweiniger A Lange J Werner A. Cognitive impairment and medial temporal lobe structure in young adults with a depressive episode. J Affect Disord. (2018) 237:112–7. 10.1016/j.jad.2018.05.015 29803901

[54] Papmeyer M Giles S Sussmann JE Kielty S Stewart T Lawrie SM. Cortical thickness in individuals at high familial risk of mood disorders as they develop major depressive disorder. Biol Psychiatry. (2015) 78:58–66. 10.1016/j.biopsych.2014.10.018 25534753

[55] Whelan CD Altmann A Botia JA Jahanshad N Hibar DP Absil J. Structural brain abnormalities in the common epilepsies assessed in a worldwide ENIGMA study. Brain. (2018) 141:391–408. 10.1093/brain/awx341 29365066 PMC5837616

[56] Peng D Shi F Li G Fralick D Shen T Qiu M Surface vulnerability of cerebral cortex to major depressive disorder. PLoS ONE. (2015) 10:e0120704 10.1371/journal.pone.0120704 25793287 PMC4368815

[57] Goldberg II Harel M Malach R. When the brain loses its self: prefrontal inactivation during sensorimotor processing. Neuron. (2006) 50:329–39. 10.1016/j.neuron.2006.03.015 16630842

[58] Fried I Wilson CL MacDonald KA Behnke EJ. Electric current stimulates laughter. Nature. (1998) 391:650. 10.1038/35536 9490408

