# Supplementary material for: Major Depressive Disorder Associated With Reduced Cortical Thickness in Women With Temporal Lobe Epilepsy
Source: Front Neurol. 2020 Jan 17;10:1398. doi: 10.3389/fneur.2019.01398 (PMC6979005; doi:10.3389/fneur.2019.01398)
Supplement: Supplementary file 1 [file Table_1.docx]

***Supplementary Material***

Table S1:

| Table S1. Pearson’s partial correlations between the *MDD-without-epilepsy* group (n=15) and the BDI-II scores revealed 24 significant cortical thickness areas. | | | |
| --- | --- | --- | --- |
| Regions | Cortical Thickness areas / gyrus / cortex | *r* (Pearson’s partial correlation) | *p*-value |
| Frontal lobe | Ipsilateral caudal middle frontal | -0.63 | <0.01 |
|  | Contralateral caudal middle frontal | -0.68 | <0.01 |
|  | Ipsilateral frontal pole | -0.67 | <0.01 |
|  | Ipsilateral lateral orbitofrontal | -0.58 | 0.042 |
|  | Ipsilateral medial orbitofrontal | -0.57 | 0.017 |
|  | Contralateral medial orbitofrontal | -0.57 | 0.016 |
|  | Ipsilateral pars triangularis | -0.69 | <0.01 |
|  | Ipsilateral rostral middle frontal | -0.65 | <0.01 |
|  | Contralateral rostral middle frontal | -0.68 | <0.01 |
|  | Ipsilateral superior frontal | -0.58 | 0.015 |
|  | Contralateral superior frontal | -0.53 | 0.025 |
| Temporal lobe | Ipsilateral fusiform | -0.58 | 0.016 |
|  | Ipsilateral transverse temporal | -0.57 | 0.017 |
| Cingulate gyrus | Ipsilateral caudal anterior cingulate | -0.51 | 0.031 |
|  | Ipsilateral rostral anterior cingulate | -0.51 | 0.030 |
|  | Contralateral rostral anterior cingulate | -0.52 | 0.027 |
| Occipital lobe | Ipsilateral cuneus | -0.61 | 0.011 |
|  | Contralateral lingual | -0.69 | <0.01 |
|  | Ipsilateral pericalcarine | -0.65 | <0.01 |
|  | Contralateral pericalcarine | -0.58 | 0.016 |
| Parietal lobe | Ipsilateral inferior parietal | -0.65 | <0.01 |
|  | Ipsilateral supramarginal | -0.51 | 0.032 |
| Insula lobe | Ipsilateral insula | -0.51 | 0.037 |
|  | Contralateral insula | -0.53 | 0.037 |

Footnote Table S1: *MDD: Major Depressive Disorder; BDI: Beck depression inventory; n= number of participants.*
